# Natural farming improves crop yield in SE India when compared to conventional or organic systems by enhancing soil quality

**DOI:** 10.1007/s13593-023-00884-x

**Published:** 2023-03-23

**Authors:** Sarah Duddigan, Liz J. Shaw, Tom Sizmur, Dharmendar Gogu, Zakir Hussain, Kiranmai Jirra, Hamika Kaliki, Rahul Sanka, Mohammad Sohail, Reshma Soma, Vijay Thallam, Haripriya Vattikuti, Chris D. Collins

**Affiliations:** 1 Soil Research Centre and Department of Geography and Environmental Science, University of Reading, Reading, Berkshire UK; 2 Rythu Sadhikara Samstha (RySS), Guntur, Andhra Pradesh India

**Keywords:** Zero budget natural farming, ZBNF, Organic farming, Conventional agriculture

## Abstract

Zero Budget Natural Farming (ZBNF) is a grassroot agrarian movement and a state backed extension in Andhra Pradesh, and has been claimed to potentially meet the twin goals of global food security and environmental conservation. However, there is a lack of statistically evaluated data to support assertions of yield benefits of ZBNF compared to organic or conventional alternatives, or to mechanistically account for them. In order to fill this gap, controlled field experiments were established in twenty-eight farms across six districts, spanning over 800 km, over three cropping seasons. In these experiments, we compared ZBNF (no synthetic pesticides or fertilisers, home-made inputs comprising *desi* cow dung and urine with mulch) to conventional (synthetic fertilisers and pesticides) and organic (no synthetic pesticides or fertilisers, no mulch, purchased organic inputs, e.g. farmyard manure and vermicompost) treatments, all with no tillage. Comparisons were made in terms of yield, soil pH, temperature, moisture content, nutrient content and earthworm abundance. Our data shows that yield was significantly higher in the ZBNF treatment (*z* score = 0.58 ± 0.08), than the organic (*z*= −0.34 ± 0.06) or conventional (−0.24 ± 0.07) treatment when all farm experiments were analysed together. However, the efficacy of the ZBNF treatment was context specific and varied according to district and the crop in question. The ZBNF yield benefit is likely attributed to mulching, generating a cooler soil, with a higher moisture content and a larger earthworm population. There were no significant differences between ZBNF and the conventional treatment in the majority of nutrients. This is a particularly important observation, as intensive use of synthetic pesticides and fertilisers comes with a number of associated risks to farmers’ finances, human health, greenhouse gas emissions, biodiversity loss and environmental pollution. However, long-term field and landscape scale trials are needed to corroborate these initial observations.

**Supplementary Information:**

The online version contains supplementary material available at 10.1007/s13593-023-00884-x.

## Introduction

The arable land area (approximately 159 Mha) in India supports the second largest volume of agricultural production in the world. This production contributes more than 15% to the national gross domestic product making it one of the most important sectors in India (Yadav et al. 2021). There has been a recognition that the green revolution, with its associated intensification of synthetic fertiliser and pesticide use, has increased crop yields but resulted in negative environmental (e.g. reduced water quality), health (exposure to toxic chemicals) and economic (farmers trapped in a cycle of debt) impacts (Agoramoorthy 2008; Bhattacharyya et al. 2015; Connor and Mínguez 2012; Mariappan and Zhou 2019; Pimentel 1996; UN 2015). Furthermore, the affordability and availability of synthetic inputs could be at risk as a result of rising natural gas and coal prices, sanctions and export restrictions and uncertainty around Indian fertiliser subsidies (World Bank Group 2022). It has been acknowledged by the United Nations (UN) that agricultural systems ‘working with nature’, that are adaptive to change and resilient, whilst minimising environmental impacts, are critical to eliminate hunger and malnutrition (UNEP 2021). Therefore, transition to these systems could contribute to the attainment of the UN Sustainable Development Goal 2 (SDG2)–Zero Hunger. Several such systems have been developed as sustainable alternatives to high input conventional farming (Willer and Lernoud 2017) including organic farming and Zero Budget Natural Farming (ZBNF).

Hundreds of thousands of farms in India are now certified as organic, with Sikkim (NE India) being declared the first all-organic certified state in the world (Meek and Anderson 2020). In principle, organic farming has the potential to reduce the environmental impact of farming through reduced use of synthetic fertilisers and pesticides, compared to conventional agriculture, but can result in a reduction in crop yield (Ponisio et al. 2015) and lower temporal yield stability (Knapp and van der Heijden 2018). Furthermore, the escalating economic and political crisis in Sri Lanka has been attributed to the unsuccessful transition to organic agriculture and blanket ban on agro-chemicals, despite there being other contributing factors (de Guzman 2022). These limitations have led to critics raising the question of whether organic farming can feed the world sustainably and without expansion of croplands into natural ecosystems (Kirchmann et al. 2008; Röös et al. 2018). Increasing the cropped area is undesirable, and the potential is limited in densely populated countries such as India (Bruinsma 2003). In addition, the socio-economic impacts associated with conventional farming may not be alleviated by organic farming in India. Large agri-businesses exert a strong control over the market for organic food, fertilisers and seeds (Bhattacharya 2017), and organic farming practices are codified in regulatory and third-party certification that can become disaggregated from the underpinning environmental principles upon which they were originally conceived (Meek and Anderson 2020; Seufert et al. 2017). Codification and commercialisation of organic farming consequently favour larger farming enterprises, leaving smallholders disadvantaged and unable to access premiums for organic produce (Panneerselvam et al. 2011).

ZBNF is a grassroots agrarian movement which is low-cost and based on locally sourced home-made amendments. ZBNF, therefore, does not rely on the use of agrochemicals or agribusiness, and it is expected to be able to achieve the twin goals of global food security and conservation of the environment (RySS 2020). In Andhra Pradesh, a state in southeast India, ZNBF (more recently referred to as Andhra Pradesh Community-Managed Natural Farming, or APCNF) has been adopted enthusiastically. The Andhra Pradesh Department of Agriculture is promoting the adoption of ZBNF through the ‘not for profit’ organisation Rythu Sadhikara Samstha (RySS). Around 580,000 farmers were engaged in ZBNF practices by 2020 (RySS 2020), and the local government plans to scale this up to 6 million farmers (Tripathi et al. 2018). It has been estimated that if ZBNF covered 25% of the total crop area in Andhra Pradesh, USD 70 million would be saved in fertiliser subsidies every year (Gupta et al. 2020). There are parallels between ZBNF and conservation agriculture in terms of the adoption of reduced tillage, application of crop residues and intercropping to reduce soil disturbance (Ravisankar et al. 2020). However, what sets ZBNF apart is the combination of these practises with unique home-made amendments. The amendments commonly used in ZBNF are as follows:

1. *Bijamrita*: a seed treatment applied either as a seed coating before sowing, or a root dip before transplanting. Common ingredients include *desi* cow dung and urine, CaCO_3_ and water
2. *Jjiwamrita*: inoculum. Can be in solid form, usually applied as a top dressing, or in liquid form as a top dressing or foliar spray. Ingredients can include *desi* cow dung and urine, jaggery (unrefined cane sugar), gram (legume) flour and topsoil from a native ‘virgin’ soil (uncontaminated soil)
3. *Achhadana*: mulching using cover crops or dry crop residues applied to the soil surface. Examples include paddy straw and groundnut husks (Ghosh 2019; Keerthi et al. 2018)

Adoption of ZBNF has been reported to increase yields in 79% of farmers surveyed (*n*=97) in Karnataka (Khadse et al. 2018), and 88% of farmers surveyed (*n* = 1614) in Andhra Pradesh (Bharucha et al. 2020) compared to ‘non-ZBNF’ management techniques. ZBNF inputs have also been observed to increase growth and yield of chilli (Gangadhar et al. 2020), peppers (Boraiah et al. 2017), rice, groundnut (Bharucha et al. 2020), maize (Vinay et al. 2020) banana, gram legumes (Galab et al. 2019) and cotton (Korav et al. 2020) compared to non-ZBNF agricultural practices. However, these studies do not always include statistical analysis to support their conclusions and do not always describe what they define as ‘non-ZBNF’. They also frequently refer to yield of total biomass rather than the economic yield. Furthermore, there is also often a lack of supporting data to mechanistically account for the benefits ZBNF can provide such as soil nutrient and moisture data. Anecdotal evidence, therefore, needs to be supported by controlled, replicated field trials (Smith et al. 2020). ZBNF performance also seems to vary in different locations (Biswas 2020) so experiments need to be conducted across the range of contexts where ZBNF is targeted. Initial work in controlled field experiments for a single season in Andhra Pradesh suggested that converting to ZBNF practices does not result in a yield penalty when compared to organic and conventional alternatives (Duddigan et al. 2022). However, there is currently a lack of supporting biophysical evidence to provide a mechanistic explanation for this finding, and whether these effects persist over multiple seasons. The efficacy of ZBNF amendments is considered to occur due to a number of key principles, put forward during workshop discussions [described in (Duddigan et al. 2022)]. Workshop participants asserted the following principles:

1. *Enhanced water holding capacity*: ZBNF practices increase soil organic matter formation which in turn leads to higher water retention.
2. *All required nutrients are in the soil*: with appropriate microbial addition in ZBNF, yields can be maintained without addition of fertiliser.
3. *Enhanced biological activity*: ZBNF practices stimulate soil biological activity, and greater earthworm populations are an indicator of this.

**Fig. 1:**
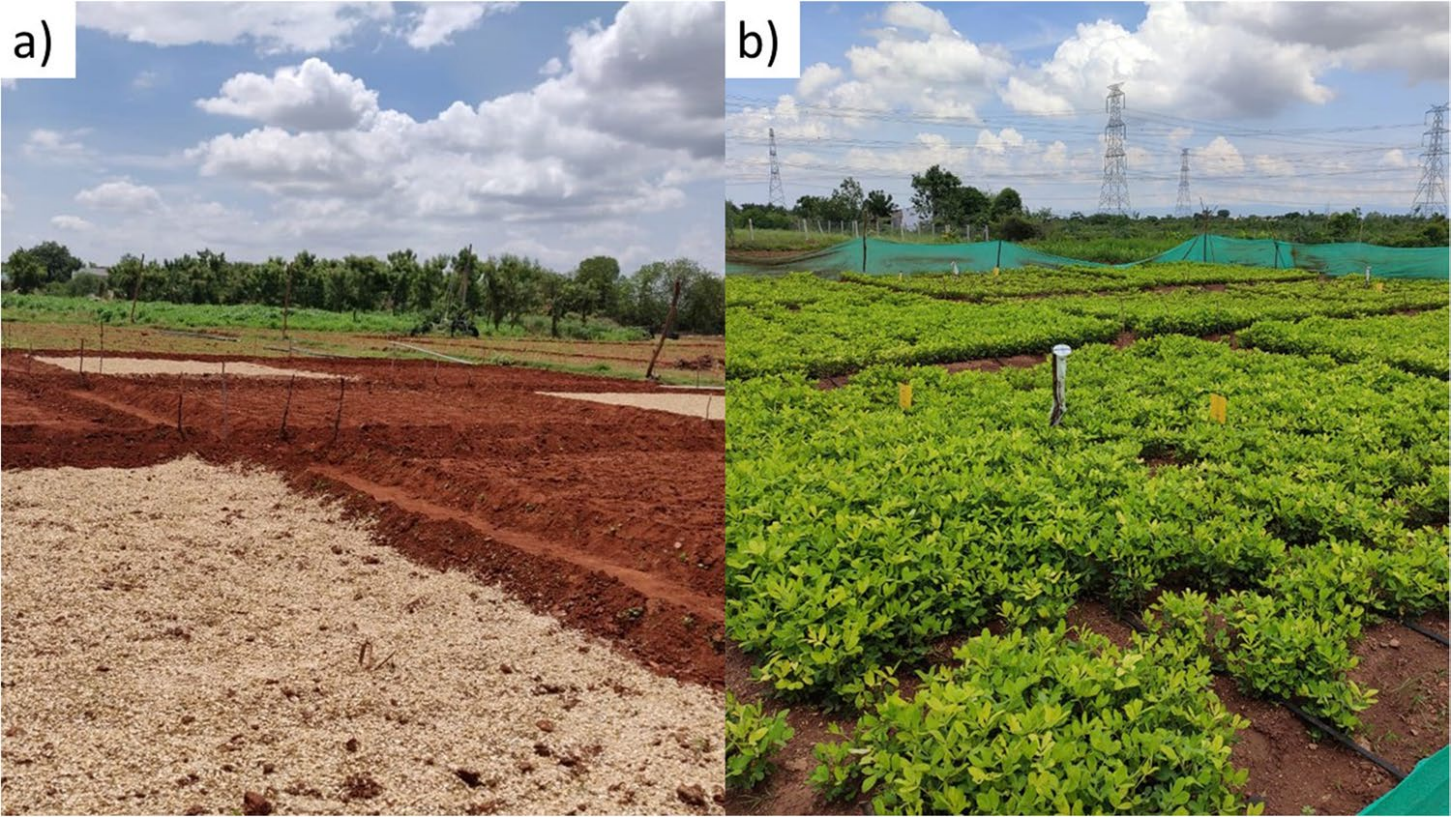
Two example field experiments comparing Zero Budget Natural Farming (ZBNF) to conventional and organic alternatives. **a** Before sowing (mulch on ZBNF treatment plots). **b** with crops established (yellow sticky traps on ZBNF treatment plots). Photo credit: **a** Ramyasree Reddymalli (RySS, Prakasam District) and **b** Lakshmi Bhairava Kumar (RySS, Anantapur District).

Using the proposed key principles above as a framework to test our hypotheses, experimental design and measurements, we aimed to examine the differences in soil physico-chemical characteristics under ZBNF, organic and conventional farming systems in replicated field experiments (Fig. 1), over three seasons, in twenty-eight farms across six geo-climatically contrasting districts of Andhra Pradesh, India.

## Materials and methods

### Site description and experimental design

Full details of the site description (with maps) and experimental layout of the field experiments can be found in Duddigan et al. (2022). The most dominant soil types in Andhra Pradesh, South Eastern India, are Alfisols and Vertisols, which account for more than 90% of the total cultivatable area of the state (Rao et al. 2013).

Field experiments were established on twenty-eight farms across Andhra Pradesh between June 2019 and June 2020. The farms were spread across six districts in Andhra Pradesh (Anantapur, Kadapa, Krishna, Nellore, Prakasam and Visakhapatnam), representing different agro-climatic zones. Ranging from the cooler, high-rainfall Northern montane (Visakhapatnam), through the lowland valley of the River Krishna (Krishna), to the warmer coastal Southern districts which abut the Bay of Bengal (Prakasam, Nellore), moving inland (Kadapa) to the scarce rainfall zone (Anantapur). A map of the farm locations can be found in the supplementary information (Figure S1). Experiments were conducted during the three major cropping seasons: (1) the *Kharif* (monsoon) season of 2019 (June–November), (2) the cooler drier *Rabi* (winter) season of 2019–2020 (Dec–June), and (3) the Kharif season of 2020. Three of the farms participated in all three seasons, fifteen of the farms conducted experiments in two of the three seasons and the remaining ten participated for just one season (Table 1). It was our original intention that all farm experiments would participate for all three seasons. However, logistical constraints resulting from the Covid-19 pandemic meant that this was not possible. Despite this, to our knowledge, this is the most extensive on the ground assessment of ZBNF performance in the region to date.

**Table 1:** Locations of participant farms and crops grown during experiment. ^*^No experiment run—farm did not participate in this season. ^**^Experiment was run but yield data was unavailable due to loss of the product during storage, e.g. pests or theft. All other data is available for the experiment.

District	Farm	Latitude (^o^)	Longitude (^o^)	Season 1 Kharif crop	Season 2 Rabi crop	Season 3 Kharif crop
Anantapur	A1	14.251	77.012	Groundnut	Chilli	No experiment^*^
	A2	13.901	78.009	Groundnut	Aubergine	No experiment^*^
	A3	14.457	77.217	Groundnut	Tomato	Groundnut
Kadapa	Ka1	14.849	78.792	Tomato	Chilli	No experiment^*^
	Ka2	14.046	78.519	Chilli	Groundnut	No experiment^*^
	Ka3	14.428	78.695	Groundnut	Tomato	Groundnut
	Ka4	14.845	78.946	Maize—no yield data^**^	Groundnut	Maize
	Ka5	14.011	78.554	No experiment^*^	No experiment^*^	Groundnut
Krishna	Kr1	16.331	80.931	Okra	Tomato	No experiment^*^
	Kr2	16.052	80.912	Okra	Sesame	No experiment^*^
	Kr3	16.436	80.938	Aubergine	No experiment^*^	No experiment^*^
	Kr4	16.684	80.784	Tomato	Sesame	No experiment^*^
	Kr5	16.622	80.904	No experiment^*^	Sesame	Okra
Nellore	N1	14.688	79.853	Okra	No experiment^*^	No experiment^*^
	N2	14.569	80.041	Okra	Green gram	No experiment^*^
	N3	14.713	79.988	Groundnut	No experiment^*^	No experiment^*^
	N4	14.685	79.228	Okra	No experiment^*^	No experiment^*^
	N5	14.543	79.912	Millet—no yield data^**^	Green gram	No experiment^*^
Prakasam	P1	15.659	80.119	Cluster bean	Chickpea	No experiment^*^
	P2	16.113	79.929	Cluster bean	Chickpea—no yield data^**^	No experiment^*^
	P3	15.427	79.971	Okra	No experiment^*^	No experiment^*^
	P4	15.231	79.992	Aubergine	Groundnut	No experiment^*^
	P5	15.695	79.909	No experiment^*^	Chickpea	No experiment^*^
Visakhapatnam	V1	18.039	82.686	Radish	No experiment^*^	No experiment^*^
	V2	18.001	83.375	Okra	No experiment^*^	No experiment^*^
	V3	18.187	82.672	Cluster bean—no yield data^**^	No experiment^*^	No experiment^*^
	V4	18.000	83.379	No experiment^*^	Carrot	No experiment^*^
	V5	17.952	82.876	No experiment^*^	Groundnut	Black gram

The same experimental design was applied on each farm, which consisted of three treatments (ZNBF, organic, conventional) applied to 6 × 6 m plots, replicated three times in a Latin square design (3 treatments × 3 replicates = 9 plots). In general, treatments consisted of (i) fungicide or insecticide seed treatment (e.g. Thiaram, Mancozeb and Imidacloprid) and fertilisers such as urea, diammonium phosphate (DAP) and potash in the *conventional* treatment; (ii) *Trichoderma* seed treatment and farmyard manure, vermicompost and biofertiliser application in the *organic* treatment; and (iii) Bijamrita seed treatment, Jiwamrita (solid and liquid) and locally sourced organic mulch application in the *ZBNF* treatment. The exact amendments and application rates varied according to the crop being grown; detailed growing protocols for each crop under each treatment can be found in Duddigan et al. (2022). Crop selection for each experiment was based on suitability for the district and local trends (i.e. what neighbouring farms were growing), to be representative of local practice. As a result, crop selection was often confounded with district. Crops were hand sown/transplanted according to the spacing outlined in the growing protocol (details in Duddigan et al. 2022) and grown as a monocrop. The field experiment was not tilled after plots were laid out, in any treatment. Due to the size of the plots, a tillage regime was not possible.

Pest and pathogen management techniques are detailed in Duddigan et al. (2022) and varied depending on the pathogen in question. Briefly, the conventional treatment consisted of chemical pesticides such as dimethoate (insecticide) and copper oxychloride (fungicide). The organic treatment used insect traps (grease coated bottles, yellow sticky plates, etc.) and/ or purchased neem oil in place of chemical insecticides, and microbial inoculants (e.g. *Trichoderma* or *Pseudomonas* sp.) in place of fungicides. The ZBNF treatment largely used insect traps, not chemical insecticides, but also used homemade ‘Neemasthram’ (cow dung, cow urine, neem seeds and leaves as well as other bitter tasting leaves available locally (e.g. castor)) and ‘Agnasthram’ (cow urine, neem leaves, tobacco leaves, chillies and garlic) in place of purchased neem oil (Kumar et al. 2019) and liquid Jiwamrita as a microbial inoculant.

Experiments were implemented and managed by RySS personnel designated as *Natural Farming Fellows* (*NFFs*)—graduates with bachelor degrees in an agricultural related subject, usually from an agricultural college. One NFF was responsible for the management of, and collection of data from, an individual experiment, approximately five per district.

### Soil sampling 

Soils were sampled three times per season: an *initial* sample taken before amendments were applied; a *mid-season* sample taken halfway through the growth cycle of the crop; and a *post-harvest* sample taken after all product and biomass has been harvested. Five soil samples were taken (0–10 cm depth) from the central 4 m × 4 m (to avoid boundary effects) in each plot in a ‘W’ formation. These were then homogenised to form one composite sample per plot for each sampling occasion.

### Soil nutrient analysis

All analyses were conducted according to Ramana Reddy et al. (2012) by the Regional Agricultural Research Station at Acharya N.G. Ranga Agricultural University (Tirupati, Andhra Pradesh). Brief methods can be found in Table 2.

**Table 2:** Laboratory soil analysis methods.

Parameter	Method	Units
Soil pH	1:2 soil to water suspension, probe	
Electrical conductivity (EC)	1:2 soil to water suspension, probe	dSm^−1^
Organic carbon (OC)	Wet digestion method (Walkley and Black 1934)	%
Extractable N	Alkaline permanganate extraction (Subbiah and Asija 1956)	kg ha^−1^
Extractable P_2_O_5_	Olsen P extraction (Olsen et al. 1954)	kg ha^−1^
Extractable K_2_O	Ammonium acetate extraction (Merwin and Peech 1951)	kg ha^−1^
Extractable Cu, Fe, Mn and Zn	DTPA extraction (Lindsay and Norvell 1978)	mg kg^−1^

### Yield

Yield was considered as the mass of produce obtained from each plot, as it would be taken to market, rather than whole plant biomass. For example, in the case of fresh vegetables, this was fresh biomass of vegetables after they were picked, and in the case of groundnut, this was the dry mass of kernels. This decision was made with stakeholders in mind, as the mass of product that can be taken to market is easy to communicate to policymakers and farmers.

### Field measurements

Field measurements were intentionally simple and robust to preclude the need for sophisticated equipment. This ensured equipment could be sourced locally, and measurements could be conducted effectively with a small period of training. The majority of measurements (soil temperature, moisture, infiltration rate, bulk density and earthworm abundance) were measured three times during each growing season at the same time that soil samples were collected: initial, mid-season, and post-harvest. Every care was taken not to sample from areas that had been disturbed by previous sampling.

#### Soil moisture and temperature

Soil moisture was measured with a moisture metre (Model PMS-714, Lutron Electronic Enterprise Co., Ltd., Taiwan) and soil temperature with a pen type plastic digital thermometer (Model DT-2, HTC Instruments, Mumbai); both probes were inserted to a depth of 5–10 cm.

#### Infiltration rate

Infiltration rate was measured in the centre of each plot with a piece of PVC pipe (c. 10 cm diameter × 20 cm) with two markings 2.5 cm apart (the first 5 cm from the top of the pipe and the second 7.5 cm from the top). Using a flat piece of wood and a mallet, the pipe was driven 4 cm into the ground. A plastic bag/sheet was then placed in the bottom of the pipe (to protect the soil from capping when the water was poured in). Water was then poured into the pipe to around the halfway mark, before the plastic was removed, and then the pipe was filled to the brim. The water was left to infiltrate into the soil until the water reached the first mark (5 cm down), when a stopwatch was started. The stopwatch was stopped when the water level reached the second marker (7.5 cm down). Infiltration rate was calculated in m/s.

#### Bulk density

The bulk density of the top 5 cm of soil was measured in the centre of each plot using a simple cylinder and driving tool method. Samples were weighed, left to dry in the sun for at least 5 days and then weighed again to obtain a dry bulk density in g cm^−3^.

#### Earthworm abundance

A single 20 × 20 × 20 cm soil block was excavated from the centre of each plot, and the soil was hand sorted to remove any earthworms in the block. All earthworms were counted, and when a balance was available (not all NFFs owned one), earthworms were cleaned of any soil particles and weighed, before being returned to the field. This earthworm count was then used to estimate total earthworm abundance per m^3^.

#### Plant biometrics

Plant biometrics were measured on five plants per plot, but the measurements that were taken depended on the crop selected. Fruiting crops such as tomato, aubergine and okra had all fruits removed from each of the five plants at harvest, where they were counted and weighed to give a ‘per plant’ yield. Legumes such as green gram and chickpea had all pods removed on 5 plants, and the pods were counted and weighed first; then, the pulses were removed and weighed to give a ‘per plant’ yield. In the case of groundnut, in addition to pod and pulse (kernel) measurements, pods were also categorised as mature or immature, judged by colour development and kernel development, as per FAO guidelines (Nautiyal 2002). Regardless of the crop in question, plant height was measured just before harvest. Dry biomass of all 5 plants after harvest was also measured for all crops.

### Statistical analysis

For yield and plant biometric data, a restricted maximum likelihood (REML) mixed effects model, with interactions and Tukey’s post hoc testing, was used (Table 3). District, treatment and crop were classified as fixed factors, and farm as a random factor, nested within district. A number of the crops selected were used in one or two farms, without repetition across districts or seasons. Therefore, we also categorised crops according whether they were a legume or not and included this as an analytical factor, to examine whether there were any general interactions for any variables between treatment and whether they were a legume or not.

**Table 3:** Summary of yield REML mixed effects model with treatment, district, season and crop variety as factors.

Factor	Type	Number of levels	Levels
District	Fixed	6	Anantapur (A), Kadapa (Ka), Krishna (Kr), Nellore (N), Prakasam (P) and Visakhapatnam (V)
Farm	Random (nested in district)	28	A1, A2, A3, Ka1, Ka2, Ka3, Ka4, Ka5, Kr1, Kr2, Kr3, Kr4, Kr5, N1, N2, N3, N4, N5, P1, P2, P3, P4, P5, V1, V2, V3, V4 and V5
Treatment	Fixed	3	Conventional, organic and ZBNF
Legume	Fixed	2	Legume and non-legume
Crop	Fixed (nested in legume)	13	Aubergine, black gram, carrot, chickpea, chilli, cluster bean, green gram, groundnut, maize, okra, radish, sesame and tomato
Season	Fixed	3	1 (Kharif), 2 (Rabi) and 3 (Kharif)
Treatment × district	Interaction		
Treatment × legume	Interaction		
Treatment × crop	Interaction		
Treatment × season	Interaction		

For variables where data was collected more than once in a season (initial, mid-season and post-harvest), a repeated measures analysis of variance (ANOVA) with least significant difference (LSD) post hoc testing was performed. The treatment factors for repeated measures were district and treatment (conventional, organic, ZBNF) with interactions. The block structure was farm and plot number, and the time points were the point in the season (initial, mid-season, post-harvest). A separate repeated measure was conducted for each season to allow for the fact that only three farms participated for all three seasons (Table 1). In order to examine select variables (e.g. extractable nitrogen) in more detail, the only three farms that participated for all three season (farm A3, Ka3 and Ka4, Table 1) were singled out for an independent repeated measures ANOVA which combined all three seasons.

Yield data was* z* transformed (Eq. 1) before being analysed with the mixed effects model. Equation 1 shows the *z* score transformation.

1 $$z=\frac{{x}_{i} - \overline{x}}{S }$$

where *z* is normalised yield for a single plot, ${x}_{i}$ is the plot yield for the single plot, $\overline{x }$ is the mean yield of all 9 plots of the given farm experiment and *S* is the standard deviation of the yield of all 9 plots on the given farm experiment. Therefore, if a plot yield is equal to the mean yield of all 9 plots on a given experiment, then *z*=0. If the plot yield is below the mean yield of all 9 plots, then *z*<0. Finally, if the plot yield is above the mean yield of all 9 plots, then *z*>0.

As a result of *z* transformation, the mean for each district, and crop, was zero, and thus, there was no effect size resulting from district or crop selected in this model. This compromise was deemed acceptable because district and crop selected are often confounded and the aim of our research was to examine the treatment effect of farming practices (i.e. contrast conventional, organic and ZBNF), rather than district or crop type. Our interest in the district and crop selected was to investigate whether there were significant interactions between them, and treatment.

## Results and discussion

### Effect on yield

**Fig. 2:**
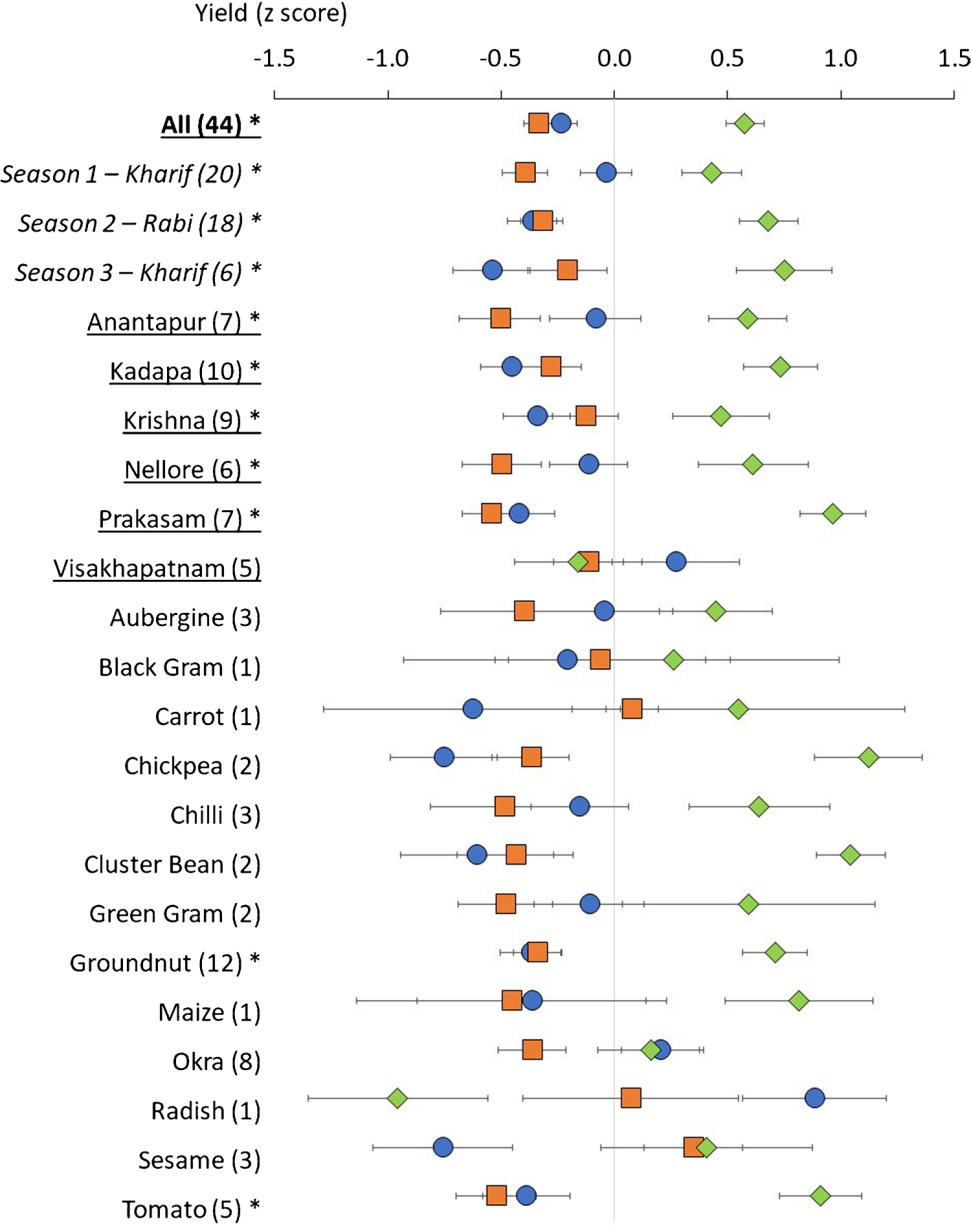
Effect of farming practice on yield (*z* transformed) of 44 field experiments (**All**) and grouped according to *season*, district and crop selected. Treatments are ZBNF (green diamond), organic (orange square) and conventional (blue circle). Numbers in brackets show the number of farms (*n*= 3 per treatment, per farm). Season 1 (Kharif) data presented in Duddigan et al. (2022). Error bars represent standard error. Groups labelled with * have a significant treatment effect (ZBNF, organic, conventional) according to a REML mixed effects model (*p *< 0.05).

Our three seasons of data suggest that adoption of ZBNF practices provides a significant yield advantage over organic and conventional alternatives. The ZBNF treatment resulted in a significantly (*p* < 0.05) higher yield, compared to organic or conventional treatments overall (Table 4 and Fig. 2). However, it is important to note that the longer-term impacts of ZBNF adoption are still unknown and will require comparative studies over an extended number of seasons to investigate. Our finding builds on initial observations of a significantly higher yield for ZBNF when compared with organic agriculture, but an equivalent performance when compared to conventional agriculture (Duddigan et al. 2022). These observations contrast with a study undertaken in Telegana state where the yield of maize in conventional farming was found to be higher than ZBNF and organic farming (Vinay et al. 2020) and a study by Galab et al. (2019) who found that rice yields were lower on ZBNF farms compared to non-ZBNF farms. Rice, however, was not grown in any of our experiments, and maize yield data was available for just one single experiment (Table 1).

**Table 4:** Results from REML mixed effects model of yield (*z* transformed) from 44 farm experiments. Significant *p*-values (< 0.05) are in bold.

Factor	*p*-value
Treatment	**<0.001**
Treatment × district	**0.002**
Treatment × legume	0.129
Treatment × crop	**0.014**
Treatment × season	0.056

ZBNF is a bottom-up transition strategy where smallholders, including tenant farmers, are key stakeholders in the process of transition (FAO et al. 2021). This immediate yield benefit observed after adopting ZBNF practices will be of particular interest to farmers on short-term land leases, as they may not be able to farm the same land every season. Andhra Pradesh has the highest percentage (42.3%) of tenant holding farmers of all the states of India, compared to the national average of 13.7% (Government of India 2015). An estimated 79% of these tenant farmers in Andhra Pradesh are either landless or own less than 1 acre of land and are therefore almost entirely dependent on leased land for their income from agriculture (Rythu Swarajya Vedika 2022). Furthermore, tenancy agreements in Andhra Pradesh can be as short as a single season (Vijayabhinandana et al. 2019), and are often on a short-term informal basis due to landowners being concerned that tenants will overstay or claim permanent occupancy of the land (Vijayabhinandana et al. 2018). However, further research is needed to examine the mid- and long-term effects of adoption of ZBNF. Particularly, if the number of tenant farms adopting ZBNF increases in the region, we might expect to see back-to-back natural farmers working the same land.

Reduced use of purchased inputs and less involvement of agri-business could also have financial benefits whilst yields are improved or maintained. It was observed that the yield *z* score for the conventional treatment reduced from season 1>2>3, whereas the organic and ZBNF mean yield *z* score increased slightly through the three seasons (Fig. 2). However, it is important to note that different farms, growing different crops, participated each season (Table 1), so this is not necessarily an indication of temporal trends in yield in the different treatments. Furthermore, there were no significant interactions between treatment and season (Table 4).

Whilst, overall, ZBNF practices, when compared as a main effect across all crops, produced a significantly higher yield, this effect was dependent on crop type (Fig. 2). There was a significant (*p* < 0.05) interaction between treatment and crop (Table 4), but a significant treatment effect was only observed for two crops, one legume (groundnut) and one non-legume (tomato). Hence, there were no significant interactions between treatment and whether the crop was a legume or not. However, groundnut and tomato were also among the most frequently grown in our experiments, providing more replicate farms to support the statistical analysis. Yield of groundnut kernels was ~30–40% higher in the ZBNF treatment (see supplementary material, Table S1). This finding is notable because groundnut is the most important oilseed crop in India (Singh et al. 2013) and covers 537,000 ha in Andhra Pradesh alone (Naik et al. 2020). To meet increased crop demands on a diminishing area of available land (16% of the land area in India remains for potential conversion to agriculture, at most), efficiency of crop production must increase (Smith et al. 2020). Therefore, methods that can improve groundnut productivity are particularly beneficial because, despite having the largest groundnut area in the world, India is not the largest producer of groundnut (Naik et al. 2020; Singh et al. 2013). Andhra Pradesh is also India’s largest producer of tomatoes, covering 167 thousand hectares (Yesdhanulla and Aparna 2018). Therefore, the benefit from the 30–40% increase in mass of fruit yield from a single plant in the ZBNF treatment compared to the organic and conventional (Table S2) could also be considerable.

Performance of ZBNF, in terms of crop yield, appears to be dependent on context, demonstrated by significant treatment × district interactions (Table 4). The northern cooler and wetter district of Visakhapatnam (Figure S1), for example, had higher yields in the conventional treatment (Fig. 2), although differences in treatments were not significant. This is in concordance with Kumar et al. (2020), who observed higher yields in conventional farms compared to natural farms in Visakhapatnam. However, our results revealed that ZBNF yield was significantly higher than both conventional and organic treatments in Prakasam, Nellore and Kadapa (Fig. 2), whereas in Krishna, ZBNF was significantly higher than the conventional treatment only, and in Anantapur, ZBNF was significantly higher than the organic treatment only. We will reflect more on regional differences in yield in later sections.

**Fig. 3:**
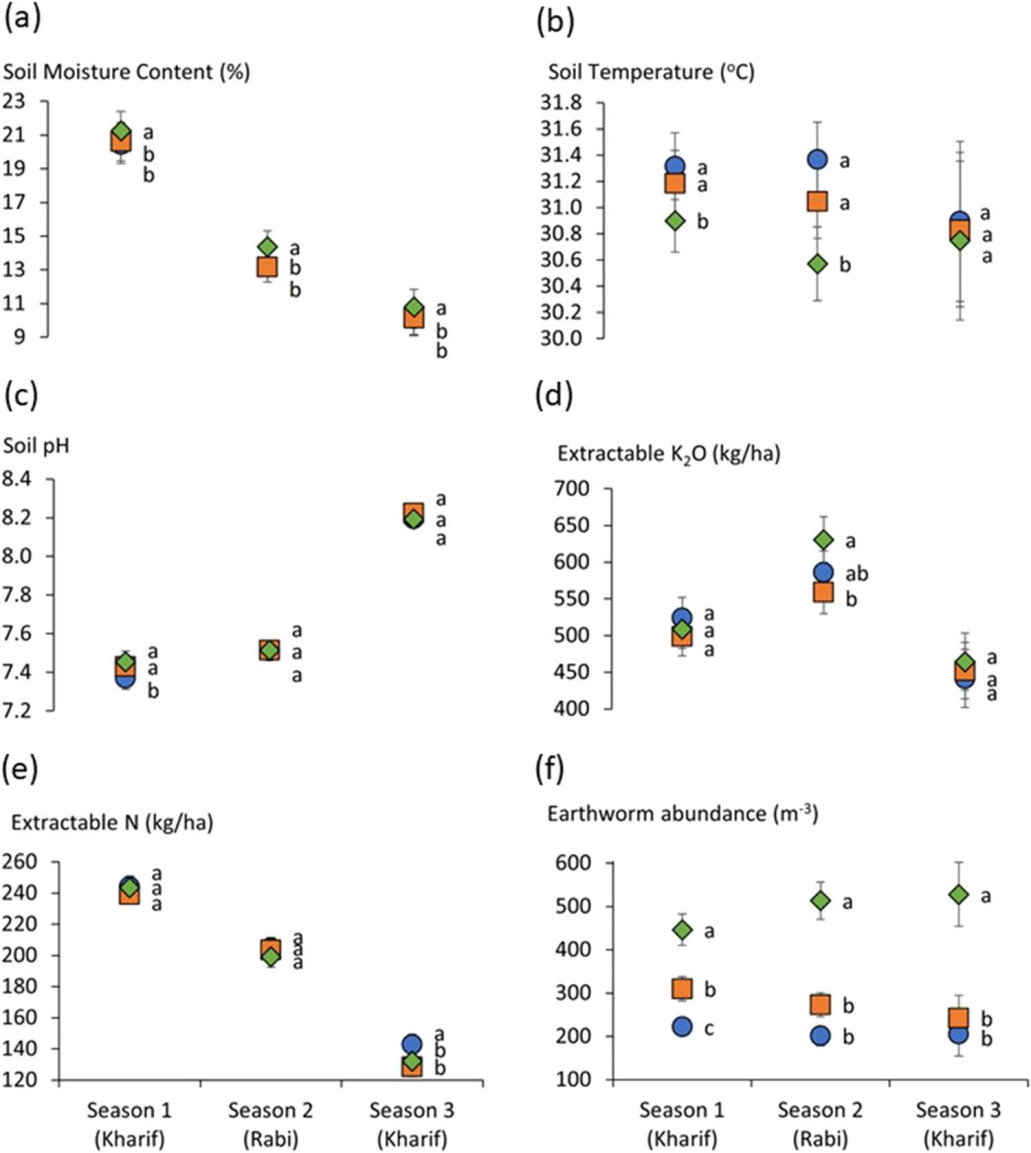
Effect of farming practice on **a** soil moisture content, **b** soil temperature, **c** soil pH, **d** extractable K_2_O, **e** extractable N and **f** total earthworm abundance across 3 seasons. Treatments are ZBNF (green diamond), organic (orange square) and conventional (blue circle). Error bars represent standard error. Treatments that share the same letter next to symbols in a particular season are not significantly different according to repeated measures ANOVA and LSD post hoc testing.

In the introduction, we outlined the key principles for the ability for ZBNF to improve crop yield, as highlighted during a stakeholder workshop. We adopted these perceived principles as hypotheses in our study and made measurements to test these hypotheses in replicated field experiments. Of all of the variables analysed, only six of them had a significant treatment effect in at least one of the seasons: (i) soil temperature, (ii) soil moisture content, (iii) soil pH, (iv) extractable K_2_O, (v) extractable N, and (vi) total earthworm abundance (Fig. 3 and Table S3). These will be discussed in the following sections. Infiltration rate also had a significant treatment effect in season 1 (see supplementary information, Table S3); however, post hoc testing did not reveal a significant difference between the treatments so is not included here. It is important to note in Fig. 3 that, because different farms participated in different seasons (Table 1), the differences between the seasons may not be a result of temporal changes in each experiment but because of a change in location of participating farms. The fact that the northern cooler and wetter region was poorly represented in season 3 would be influential in this respect. Ideally, all farms would have participated for all three seasons, but restrictions during the Covid-19 pandemic meant that this was not possible. However, the data presented here still provides valuable insights into the efficacy of ZBNF farming practices.

### ZBNF claim 1: enhanced water holding capacity

It has been suggested that ZBNF practices increase soil organic matter formation which, in turn, leads to higher water retention (Khadse and Rosset 2019). However, our findings suggest that the mulch had more of a direct effect on soil moisture maintenance than building organic matter over the timescale of study. There was no significant treatment effect on bulk density or organic carbon, suggesting that treatment has no immediate significant effect on soil organic matter content. This is to be expected as there would be a delay before belowground food webs were established, and litter derived C is stabilised into more persistent forms (Crews and Rumsey 2017; Kallenbach and Grandy 2011; Plaza et al. 2013; Stockmann et al. 2013). However, mulching with organic material in the ZBNF treatment can have immediate direct effects on regulation of soil temperature and moisture to improve crop yield (Chavan et al. 2009; Chen et al. 2007; Kader et al. 2017) through changes in albedo and reduced evaporation in arid regions (Liu et al. 2014; Tuure et al. 2021). ZBNF plots had a significantly higher soil moisture content (Fig. 3a) and subsequently lower soil temperature (Fig. 3b) compared to organic and conventional treatments. However, the difference between treatments was not significant for soil temperature in season 3 (Fig. 3b). In addition, mulching can have other benefits for crop production, such as weed suppression (Thankamani et al. 2016) and thus reduced competition for water (and nutrient) uptake with the crop. Weed cover was not quantified in this experiment, but research into this in the future would be beneficial.

There was a significant negative correlation between the initial soil moisture content of each farm site, before any treatments were applied, and mean ZBNF yield (*z* score) of the experiments first season (i.e. the first season they participated) (Spearman correlation coefficient −0.442, *p*=0.031, Figure S2). This correlation suggests that ZBNF has greater efficacy in drier farms. This finding builds on the observations in Duddigan et al. (2022) that the yield benefit of ZBNF was greatest in the hottest and driest regions of Andhra Pradesh. This phenomenon may also explain why the yield benefits of ZBNF got progressively greater through the seasons (Fig. 2), because average soil moisture content of experiments (Fig. 3a) in season 2 overall (13.6% ± 0.69) was lower than season 1 (20.8% ± 0.42) and lower still in season 3 (10.3% ± 0.40). Paddy straw mulch (commonly used in the ZBNF treatments) has been shown to improve crop growth by buffering fluctuations in soil moisture and temperature, more so than plastic, paper and dry grass (Kader et al. 2017).

There were no significant ‘per plant’ biometric treatment effects for groundnut, but there was a significant ‘per plot’ treatment effect on groundnut biometrics (Table S1). Given that seed rates were the same in all treatments, this suggests that ZBNF increased yield due to improved germination or crop establishment (i.e. more plants) rather than improving the quality or size of the individual plants. However, we do not have plant count or emergence data to support this. It has been observed that soil surface temperature controls the rate of seedling emergence in groundnut. However, it is often observed that groundnut emergence increases with increasing temperatures (Prasad et al. 2006). In addition, the optimum temperature for groundnut emergence has been suggested to be between 32 and 33°C (Leong and Ong 1983), which is higher than the average temperature observed in any of the treatments in our research. Therefore, it is more likely that increased soil moisture content is improving emergence of groundnut directly in the ZBNF treatment. Furthermore, K, which occurs in higher concentrations in the ZBNF treatment in season 2, has been shown to alleviate adverse effects of water stress on groundnut yield (Umar 2006). Groundnut is capable of rooting to depths exceeding 90 cm by 70 days after sowing and could potentially extract water to 150 to 250 cm (Black et al. 1985). Taken together, these observations suggest that increased water retention at the soil surface through mulching in the ZBNF treatment will be less important to groundnut when the plants get larger as they can exploit deeper water reserves. This concept could account for per plant biometrics having no significant difference between treatments. Tomato plants, on the other hand, benefit from light and frequent water supply throughout the growing season to improve growth, yields and fruit size (FAO 2021). This difference may account for both ‘per plant’ and ‘per plot’ biometrics being significantly higher for tomatoes in the ZBNF treatment. Particularly, a light and frequent water supply will be provided through the application of liquid Jiwamrita.

### ZBNF claim 2: all required nutrients are in the soil 

Enhanced yield by microbial inoculants has been linked, in some cases, to enhanced nutrient uptake and improved nutrient status of plants (Calvo et al. 2014). The principle put forward in the workshop discussion was that, with appropriate microbial addition in ZBNF, yields can be maintained without addition of fertiliser. It is claimed that all the nutrients a crop needs are already present in the soil, and application of beneficial microorganisms present in Jiwamrita catalyses the transformation of nutrients locked up in the soil into plant-available forms (Biswas 2020; Keerthi et al. 2018; Korav et al. 2020). Both the solid and liquid Jiwamrita are intended to act as a microbial inoculant, increasing soil biodiversity and acting as a plant ‘biostimulant’. Plant biostimulants are substances and/or microorganisms that, rather than supplying nutrients directly, aim to stimulate a plant’s natural nutrient acquisition process, thereby enhancing plant growth, increasing tolerance to unfavourable soil and environmental conditions and improving resource use efficiency (European Union 2019)

Given the claims of ZNBF in relation to promotion of plant availability of nutrients, our comparison of soil nutrient status across the ZNBF, organic and conventional treatments utilised chemical extractions intended to mimic plant nutrient uptake from labile soil nutrient pools (Table 2) and thus focussed on ‘available’ or ‘potentially available’ nutrients rather than total nutrient stocks. For P, K and micronutrients (Cu, Fe, Mn, Zn), the results suggest that nutrient availability is unaffected by treatment (Supplementary information, Table S3); with the exception of K_2_O in season 2 (Fig. 3d), there was no significant difference between treatments. This is an important observation because the conventional treatment, that used synthetic fertilisers, did not increase extractable nutrient concentrations compared to organic and ZBNF treatments. When compared to the conventional treatment, yields were indeed maintained, in the case of organic, and increased in the ZBNF treatment. There are a number of mechanisms that have been suggested to be at work in the liberation of nutrients being held in the soil after Jiwamrita application: (i) nutrient supply as a consequence of mineralisation and solubilisation activity by detrital food webs, (ii) improved plant uptake of (in particular) immobile nutrients (i.e. P and Zn) via mycorrhizal fungi and (iii) microbial production of plant growth hormones that increase root area and thus nutrient uptake from soil. However, there is insufficient evidence here to suggest that microbial additions are liberating nutrients from the soil in the ZBNF treatment, as was claimed by stakeholders in the workshop discussion. Detailed analysis of the microbially mediated processes involved in mineralisation and solubilisation of nutrients to plant available forms and nutrient uptake in these systems is needed to examine these assertions further.

It has been suggested that it is likely that ZBNF systems could be more deficient in nitrogen than conventional systems (Smith et al. 2020). In season 3 of our experiment, there was a significantly higher extractable N content in the conventional treatment than the ZBNF or organic treatments (Fig. 3e). Season 3, on the whole, had lower extractable N than season 1 or 2. However, as previously stated, different farms participated in each season so this observation is not necessarily an indication of temporal trends. In Table 5, we show the temporal trends in extractable N in the three farms that participated across all three seasons. Here, we show that, although there was significantly lower extractable N in the ZBNF treatment in the final sample taken and the initial sample in one of the farms (A3), there was no significant difference between treatments, in any of the farms, indicating that extractable N decreased in all treatments on all three farms over time. Furthermore, whilst our research focussed on the amendments used in ZBNF and crops were grown as a monocrop, intercropping is also commonplace in ZBNF, particularly with legumes. This is another possible mechanisms for N provision in ZBNF that we were not able to explore and would require closer examination in the future.

**Table 5:** Effect of farming practice on extractable N (kg/ha) in farms that participated in all three seasons. Suffix letters signify the results of repeated measures ANOVA and LSD post hoc testing. Treatments that share a lowercase letter in the same row are not significantly different for that time point. Time points that share the same uppercase letter in the same column are not significantly different for that particular treatment of each farm.

Sampling time	Conventional	Organic	ZBNF
Farm A3 (Anantapur)			
Season 1 (Kharif)—initial	267.6 ± 8.36aA	179.8 ± 39.89aA	292.7 ± 18.23aA
Season 3 (Kharif)—post-harvest	163.3 ± 7.22aA	129.7 ± 23.47aA	142.3 ± 15.07aB
Relative change^*^	−0.4 ± 0.03	−0.2 ± 0.15	−0.5 ± 0.05
Farm Ka3 (Kadapa)			
Season 1 (Kharif)—initial	192.3 ± 18.23aA	225.8 ± 14.48aA	209.1 ± 53.38aA
Season 3 (Kharif)—post-harvest	96.0 ± 14.98aA	96.0 ± 4.00aB	108.7 ± 27.42aA
Relative change^*^	−0.5 ± 0.03	−0.6 ± 0.01	−0.5 ± 0.01
Farm Ka4 (Kadapa)			
Season 1 (Kharif)—initial	250.9 ± 26.11aA	263.4 ± 31.57aA	209.1 ± 27.42aA
Season 3 (Kharif)—post-harvest	196.3 ± 8.33aA	171.3 ± 8.33aA	192.3 ± 4.33aA
Relative change^*^	−0.2 ± 0.07	−0.3 ± 0.09	0.0 ± 0.13

### ZBNF claim 3: earthworm population

The third claim put forward by ZBNF promoters is that ZBNF practices enhance the activity of soil biology, and larger earthworm populations are an indicator of this. Higher earthworm abundance has previously been observed in ZBNF fields compared to non-ZBNF fields (Bharucha et al. 2020). In our research, earthworm abundance was indeed significantly and considerably higher in the ZBNF treatment than the conventional or organic treatment in all three seasons (Fig. 3f), along with earthworm biomass (Supplementary information, Figure S3) likely a result of mulching. Crop residue, or dead mulch, retained on the soil surface can lead to higher earthworm abundance through reduced soil temperature, moisture retention and increased food resources so that the earthworms can grow and reproduce (Paoletti 1999; Turmel et al. 2015). Temperature is known to impact the behaviour, growth and density of earthworms (Al-Maliki et al. 2021); therefore, the reduced temperatures observed in the ZBNF treatment, discussed above, may benefit the earthworm community. In our research, we did not record the ecological group of the earthworms (epigeic, endogeic or anecic) collected. However, it is important to note that the effects of mulching, and the subsequent effect on soil temperature and food supply, will have varying impacts on earthworms depending on their ecological niche, with surface dwelling epigeic earthworms, for example, that do not move deeper into the profile, standing to benefit the most from surface mulching (Al-Maliki et al. 2021; Turmel et al. 2015). Applications of cow dung and Jiwamrita have also been found to increase earthworm abundance during treatment of agro-industrial waste (Veeresh and Narayana 2013). Earthworm abundance has also been observed to be higher in organic farming than conventional in semiarid northern regions of India (Suthar 2009), which we also observed in season 1. Due to the size of the plots, a tillage regime was not possible; therefore, the field experiment was not tilled after plots were laid out, in any treatment. However, it is important to highlight that, ZBNF, conventional and organic farming will have different approaches to tillage in practise, which will have additional impacts on soil biota (Crittenden et al. 2014). This will need to be considered in future research.

The increased abundance of earthworms can have a number of indirect benefits on yield through their role in nutrient cycling, plant pathogen suppression and development of soil structure, thereby influencing aeration and drainage (Blouin et al. 2013; Plaas et al. 2019; Sharma et al. 2017). A meta-analysis found that presence of earthworms in agroecosystems can lead to an average 25% increase in plant production (van Groenigen et al. 2014). Furthermore, the positive effects of earthworms were observed to be more prominent in systems where crop residues are applied/returned to the soil (van Groenigen et al. 2014), suggesting that earthworms may play a larger role in ZBNF systems that involve application of crop residues in the form of mulch.

Earthworm abundance has been recognised as a potentially useful indicator of soil quality, largely due to their sensitivity to soil disturbance (Doran and Zeiss 2000; Falco et al. 2015; Ritz et al. 2009). Research has also suggested earthworms are good indicators of some beneficial microbial functions. For example, a study in Andhra Pradesh observed that earthworms could be a vector for translocation and dispersal of mycorrhiza in groundnut (Lee et al. 1999). Earthworms can contribute to the structuring of belowground microbial communities both directly through their ingestion or indirectly though comminution of substrates and increased availability of easily assimilated substances for microbes in earthworm middens (Bohlen et al. 2002; Edwards 2004; Medina-Sauza et al. 2019). However, the extent of this influence on the microbial community is dependent on the ecological group of earthworms in question (Medina-Sauza et al. 2019). We did not record earthworm ecological group in this study, or whether earthworms were juvenile or adult; future research on the link between earthworm abundance and microbial activity would benefit from this information.

## Conclusions

The aim of this study was to provide evidence from replicated field trials to assess the performance of ZBNF compared to conventional and organic alternatives, and mechanistically account for the benefits ZBNF can provide. The three seasons of data we present here suggest that there was no yield penalty in the ZBNF treatment in any of the districts investigated, and some districts observed a yield benefit in the ZBNF treatment. We suggest that the ZBNF treatment benefits derive from higher soil moisture content, lower soil temperature and a larger earthworm population as a consequence of mulch addition. However, more research into the contribution of each of the individual ZBNF inputs (Bijamrita, solid Jiwamrita, liquid Jiwamrita and mulch) is needed to test this. Closer examination of the availability of these inputs if operated at scale will also be vital. In addition, whilst our research has focussed on the amendments used in ZBNF, there are other elements of ZBNF management in combination with the amendments that need further examination in the future, such as intercropping and reduced tillage. Initial observations that there were no significant differences between treatments in the majority of nutrients, despite ZBNF and organic treatments receiving no synthetic fertiliser inputs, is an important one if they can be replicated across Andhra Pradesh. This is a particularly important finding as intensive use of synthetic pesticides and fertilisers comes with a number of associated risks to farmer finances, human health, greenhouse gas emissions, biodiversity and environmental pollution. However, long-term field and landscape scale trials are needed to corroborate these observations if ZBNF is going to be adopted at scale.

## Supplementary Information

Below is the link to the electronic supplementary material.

Supplementary file1 (PDF 330 KB)

## Data Availability

Data is available on request.
