# Supplementary material for: Natural farming improves crop yield in SE India when compared to conventional or organic systems by enhancing soil quality
Source: Agron Sustain Dev. 2023 Mar 23;43(2):31. doi: 10.1007/s13593-023-00884-x (PMC10035491; doi:10.1007/s13593-023-00884-x)
Supplement: Supplementary file 1 — Supplementary file1 (PDF 330 KB) [file 13593_2023_884_MOESM1_ESM.pdf]

Supplementary Information

Table S1 – Groundnut biometrics. Treatments in the same row that share a lower case letter are not significantly different according to REML mixed effects model and Tukey's post hoc testing (p<0.05)

		**Mean ± S.E.**	
	**Conventional**	**Organic**	**ZBNF**
Mass kernels (kg/plot)	5.43 ± 0.83b	4.91 ± 0.59b	6.90 ± 0.94a
Dry biomass (kg/plot)	5.20 ± 0.80b	5.00 ± 0.80b	6.48 ± 1.01a
Mass pods (kg/plot)	8.19 ± 0.77b	7.84 ± 0.62b	10.03 ± 0.82a
Mass kernels (g/plant)	15.57 ± 3.18a	15.11 ± 2.93a	16.35 ± 3.09a
Dry biomass (g/plant)	47.54 ± 8.88a	46.63 ± 8.40a	51.85 ± 10.04a
Number of pods (/plant)	17.00 ± 1.73b	16.56 ± 1.61b	18.10 ± 1.91a
Mass pod (g/pod)	1.57 ± 0.27a	1.52 ± 0.29a	1.61 ± 0.29a
Proportion mature pods (mature/total pods per plant)	0.76 ±0.06a	0.73 ± 0.06a	0.81 ± 0.07a
Plant height (cm)	37.90 ± 1.47b	36.59 ± 1.40b	40.89 ± 1.45a
Number of root nodules (/plant)	86.35 ± 10.56a	81.88 ± 9.66a	82.33 ± 10.50a

Table S2 – Tomato biometrics. Treatments in the same row that share a lower case letter are not significantly different according to REML mixed effects model and Tukey's post hoc testing (p<0.05)

		**Mean ± S.E.**	
	**Conventional**	**Organic**	**ZBNF**
Mass fruits (kg/plot)	24.71 ± 2.87b	23.20 ± 2.83b	31.15 ± 2.96a
Dry biomass (kg/plot)	4.20 ± 0.40a	3.90 ± 0.37a	4.70 ± 0.38a
Number of fruits (/plant)	6.52 ± 0.60ab	6.05 ± 0.45b	7.33 ± 0.45a
Mass fruits (g/plant)	347.59 ± 44.83b	364.05 ± 39.44b	487.57 ± 58.89a
Fruit mass (g/fruit)	58.48 ± 7.61a	64.66 ± 7.50a	69.16 ± 7.70a
Dry biomass (g/plant)	47.74 ± 18.66ab	46.41 ± 17.41b	50.99 ± 18.43a
Plant height (cm)	42.99 ± 3.10a	42.48 ± 2.85a	46.43 ± 2.53a

Table S3 - Effects of farming practise on soil characteristics. Results from repeated measures ANOVA

	**Treatment *p*-values according to Repeated Measures ANOVA**		
**Variable**	**Season 1 (Kharif)**	**Season 2 (Rabi)**	**Season 3 (Kharif)**
Bulk Density	0.311	0.730	0.101
Infiltration rate	**0.038**	0.451	0.709
Soil Temperature	**<0.001**	**<0.001**	0.721
Soil Moisture	**0.006**	**<0.001**	**0.015**
pH	**0.013**	0.727	0.262
EC	0.082	0.272	0.644
OC	0.902	0.200	0.837
Extractable N	0.761	0.744	**0.010**
Extractable P_2O_5	0.734	0.062	0.958
Extractable K_2O	0.264	**0.004**	0.306
Extractable Cu	0.436	0.121	0.732
Extractable Mn	0.693	0.770	0.921
Extractable Fe	0.271	0.397	0.521
Extractable Zn	0.152	0.358	0.590
Total Earthworm Abundance	**<0.001**	**<0.001**	**<0.001**
Total Earthworm Biomass	**<0.001**	**<0.001**	**0.013**

District	Mean Annual Rainfall (mm)	Mean Annual Temperature (°C)
Anantapur	500-600	28-29
Kadapa	600-700	28-29
Krishna	800-900	28-29
Nellore	800-900	29-30
Prakasam	600-700	28-29
Visakhapatnam	1000-1100	26-27

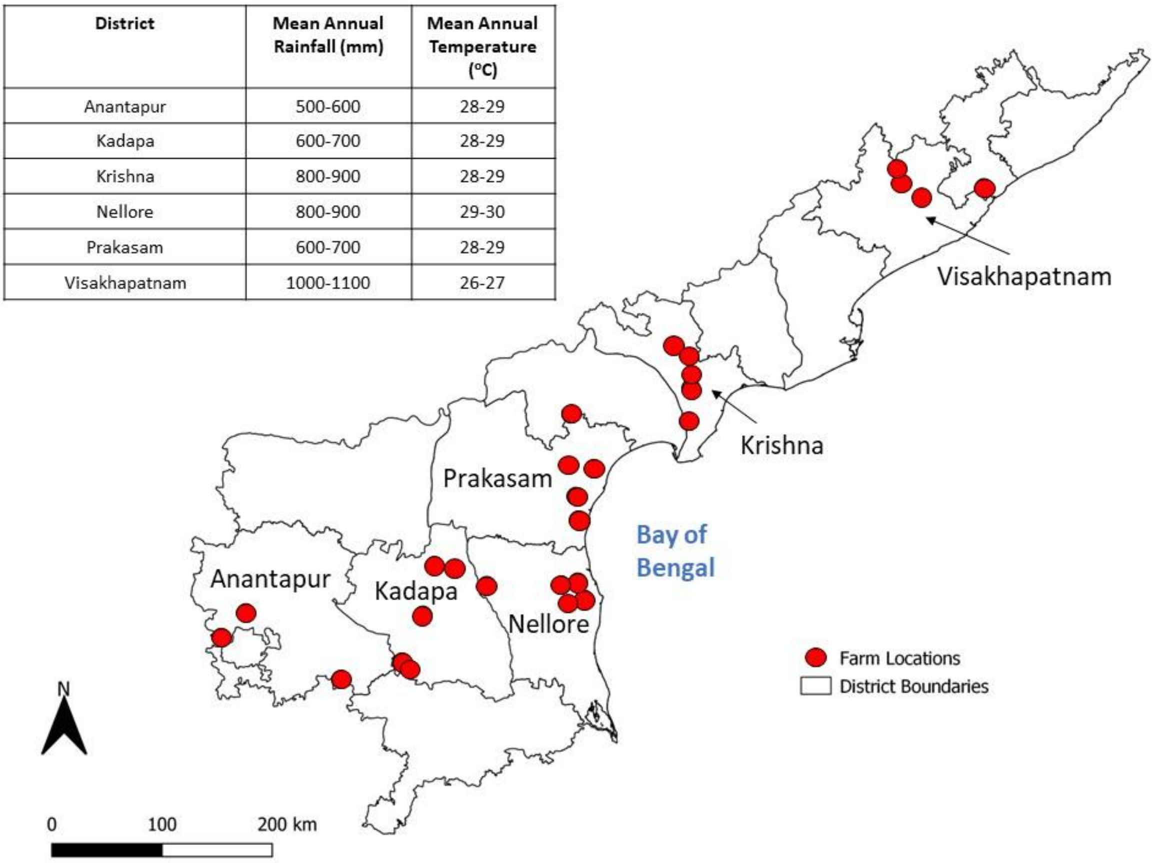

Figure S1 – Locations of experimental field sites

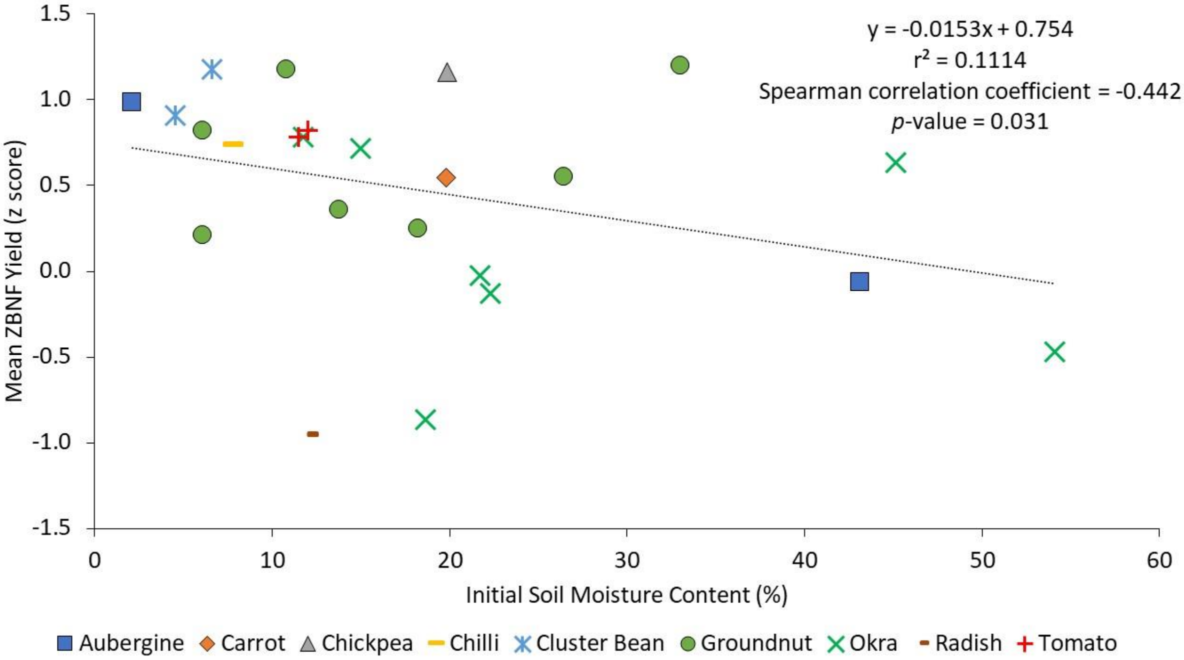

Figure S2 - Initial soil moisture content of each farm against their first seasons yield in the ZBNF treatment

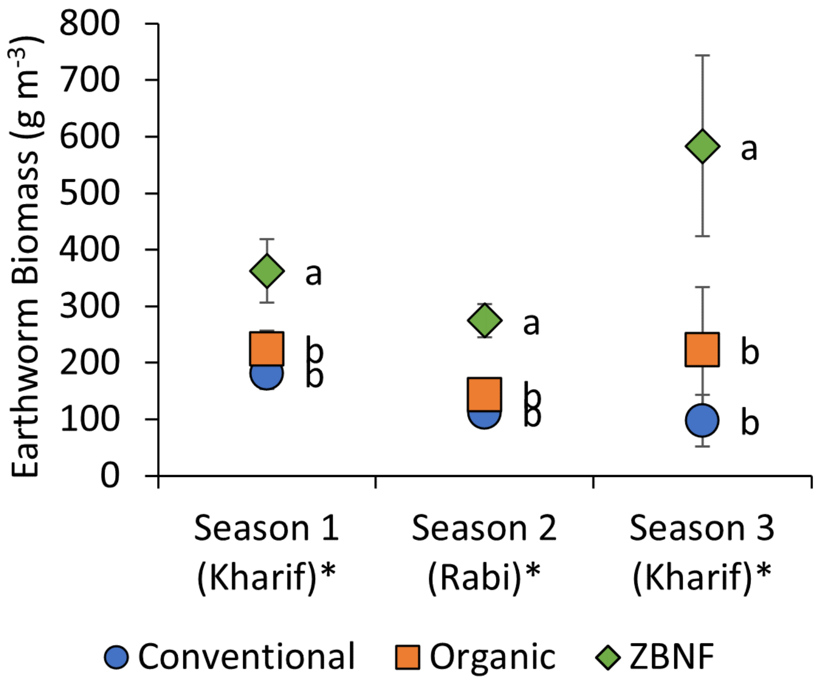

Figure S3 - Effect of farming practice on earthworm biomass across 3 seasons. Error bars represent standard error. Seasons marked with * are seasons where there was a significant treatment effect according to repeated measures ANOVA for the season, treatments that share the same letter next to symbols in a particular season are not significantly different according to LSD post-hoc testing
